# Cerebral Small Vessel Disease Burden Is Increased in Systemic Lupus Erythematosus

**DOI:** 10.1161/STROKEAHA.116.014330

**Published:** 2016-10-25

**Authors:** Stewart J. Wiseman, Mark E. Bastin, Charlotte L. Jardine, Gayle Barclay, Iona F. Hamilton, Elaine Sandeman, David Hunt, E. Nicole Amft, Susan Thomson, Jill F.F. Belch, Stuart H. Ralston, Joanna M. Wardlaw

**Affiliations:** From the Centre for Clinical Brain Sciences (S.J.W., M.E.B., C.L.J., G.B., I.F.H., E.S., D.H., J.M.W.) and Centre for Genomic and Experimental Medicine (S.H.R.), University of Edinburgh, United Kingdom; Department of Rheumatology, Western General Hospital, Edinburgh, United Kingdom (E.N.A.); and Division of Cardiovascular and Diabetes Medicine, University of Dundee, United Kingdom (S.T., J.F.F.B.).

**Keywords:** autoimmune diseases, biomarkers, C-reactive protein, inflammation, systemic lupus erythematosus

## Abstract

**Background and Purpose—:**

Systemic lupus erythematosus (SLE) increases stroke risk, but the mechanism is uncertain. This study aimed to determine the association between SLE and features on neuroimaging of cerebral small vessel disease (SVD), a risk factor for stroke.

**Methods—:**

Consecutive patients attending a clinic for SLE were recruited. All patients underwent brain magnetic resonance imaging; had blood samples taken for markers of inflammation, endothelial dysfunction, cholesterol, and autoantibodies; and underwent cognitive and psychiatric testing. The data were compared with sex- and age-matched healthy controls and patients with minor stroke. Features of SVD were measured, a total SVD score calculated, and associations sought with vascular risk factors, cognition, SLE activity, and disease duration.

**Results—:**

Fifty-one SLE patients (age: 48.8 years; SD: 14.3 years) had a greater total SVD score compared with healthy controls (1 versus 0; *P*<0.0001) and stroke patients (1 versus 0; *P*=0.02). There were higher perivascular spaces and deep white matter hyperintensity scores and more superficial brain atrophy in SLE patients versus healthy controls. Despite fewer vascular risk factors than similarly aged stroke patients, SLE patients had similar or more of some SVD features. The total SVD score was not associated with SLE activity, cognition, disease duration, or any blood measure.

**Conclusions—:**

In this data set, SLE patients had a high burden of SVD features on magnetic resonance imaging, particularly perivascular spaces. A larger longitudinal study is warranted to determine the causes of SVD features in SLE and clinical implications.

## Abstract 2

Supplemental Digital Content is available in the text.

The inflammatory autoimmune disease systemic lupus erythematosus (SLE) can involve the brain, including increasing stroke risk for reasons that remain incompletely understood.^1^

Cerebral small vessel disease (SVD) is an intrinsic disorder of the brain’s perforating arterioles.^2^ Imaging features range from asymptomatic white matter hyperintensities (WMH) and other brain imaging biomarkers^3^ of SVD such as an increase in number and size of visible perivascular spaces (PVS) to symptomatic lacunar stroke, which accounts for ≈25% of ischemic strokes. Symptoms also include cognitive impairment and dementia, depression, and balance problems.^2^

In sporadic SVD, inflammation and cell infiltrates are seen in the perforating arteriolar walls, and microglial activation is seen in the perivascular tissue on pathology.^4^ The source of the inflammation is not known, whether intrinsic or triggered by systemic processes. However, consistent with an inflammatory component, SVD-related PVS are associated with raised plasma markers of inflammation in healthy older subjects.^5^ Also, C-reactive protein (CRP), a common plasma marker of inflammation, was associated with lacunar infarcts in a recent large (n=519) study, independent of age and vascular risk factors.^6^ Thus, factors that contribute to endothelial damage, such as immune complex formation and complement activation/deposition, and occur in SLE might trigger cerebrovascular inflammation in SLE.

We hypothesized that one explanation for increased stroke risk in patients with SLE could be via the effects of systemic inflammation on cerebral small vessel integrity. Our aims were to measure and compare imaging biomarkers of SVD in patients with SLE with sex- and age-matched healthy controls and patients with minor including lacunar stroke and, in patients with SLE, compute a total burden of SVD score and determine associations with vascular risk factors, plasma biomarkers of inflammation, endothelial dysfunction, cholesterol, cognition, and clinical measures of SLE disease activity and damage.

## Methods

### Subjects

This cross-sectional brain magnetic resonance imaging (MRI) study prospectively recruited patients with SLE—including members of the Scottish Lupus Exchange Database (UK Clinical Trials ID 15489)—who attended a regional specialist clinic between April and December 2014. The clinic reviews all patients diagnosed with SLE in one health region from the point of diagnosis onward. We recruited as consecutively as possible, and SLE patients represented a wide range of SLE, being of varying disease durations and severities. All patients were seen by a consultant rheumatologist; clinics were run jointly with a neurologist and renal physician. SLE was diagnosed according to updated American College of Rheumatology 1997 criteria.^7^ The project received research ethics committee approval (South-East Scotland Research Ethics Committee 01, 14/SS/0003), and all participants gave written informed consent.

We obtained control data from healthy volunteers aged between 25 and 65 years, recruited by poster campaign at the same health region. Volunteers were recruited if they were native English speakers, were not on any long-term medication, had not been diagnosed with any chronic medical condition including diabetes mellitus or hypertension, had not undergone previous cranial surgery, and were able to undergo brain MRI. The study was approved by the Lothian Research Ethics Committee (05/S1104/45), and subjects gave written informed consent.

We also compared SLE patients to patients with first-ever minor (National Institutes of Neurological Disorders and Stroke scale <7 or nondisabling) stroke including those of small vessel (lacunar) type, recruited from the same health region via the regional stroke service. The study was approved by the South-East Scotland Research Ethics Committee 01 (09/S1101/54). A stroke specialist determined the ischemic stroke subtype (lacunar or minor cortical) using the Oxfordshire Community Stroke Project^8^ clinical classification and confirmed by MRI.

Patients with SLE and both control groups were scanned with similar sequences on the same magnetic resonance scanner (details below) that underwent regular maintenance including daily quality assurance.

### Vascular Risk Factors—SLE Patients

Medical histories including cardiovascular risk factors such as smoking status, cerebrovascular events, hypertension, and diabetes mellitus were recorded. Height and weight were measured, and body mass index calculated. We measured blood pressure 3 times (before the MRI scan, after the scan, and at the end of the study visit) and noted antihypertensive medication. We dichotomized patients as hypertensive or not and also classified them with the British Hypertension Society 6-point scale, from optimal to severe.^9^

### SLE Activity and Damage Scores

SLE disease activity was assessed by an experienced rheumatology nurse specializing in SLE, who interviewed each patient and had access to all medical data and blood results using the Systemic Lupus Erythematosus Disease Activity Index 2000 (SLEDAI-2K)^10^ and British Isles Lupus Assessment Group 2004 (BILAG)^11^ tools. Accumulated permanent damage was assessed with the Systemic Lupus International Collaborating Clinics (SLICC)^12^ tool.

### Fatigue

Fatigue was assessed using the Fatigue Severity Scale^13^ with higher scores indicating more severe fatigue. The mean (SD) from normal healthy adults in the standardization sample was 2.3 (0.7).^13^

### Cognitive and Psychiatric Assessments

We used Hospital Anxiety and Depression Scale,^14^ Montreal Cognitive Assessment,^15^ Addenbrooke’s Cognitive Examinations–Revised,^16^ and Mini Mental State Examination^17^ to assess anxiety, depression, and cognitive function. The tests were administered by the study team, not to screen for neurolupus. However, before recruitment, all SLE patients were seen at the SLE clinic that monitors for cerebral involvement in collaboration with neurology, and the 4 of 51 with neuropsychiatric systemic lupus erythematosus (NPSLE) were diagnosed by a consultant neurologist with a special interest in neurolupus.

### Magnetic Resonance Imaging

All patients and controls underwent neuroimaging at 1.5T (Signa HDx; GE, Milwaukee, WI). The following were acquired: axial T2, gradient-recalled echo, fluid-attenuated inversion recovery, sagittal T2, high-resolution coronal 3-dimensional T1 volume, and whole brain diffusion tensor MRI (please see Table I in the online-only Data Supplement for scan parameters). The diffusion tensor MRI scans were used to assess white matter microstructural integrity by measuring mean diffusivity and fractional anisotropy.

### Image Review and Visual Rating

All MRI scans were reviewed by a consultant neuroradiologist blind to all other data. Imaging features of SVD were defined per STRIVE guidelines.^3^ Deep and periventricular WMHs were coded 0 to 3 using the Fazekas^18^ scale and summed to give a total WMH score (0–6) per subject. Visible (enlarged) PVS are round (<3 mm) or linear depending on the orientation of the scan plane to the vessel^19^ and their intensity is that of cerebrospinal fluid on T2-weighted MRI. They were assessed in the basal ganglia and centrum semiovale and scored as 0 (none), 1 (1–10 PVS), 2 (11–20 PVS), 3 (21–40 PVS), and 4 (>40 PVS) using a validated scale.^19,20^ Lacunes^21^ were defined as deep infarcts, distinguished from PVS because of their larger size (3–20 mm), and their presence, including location in the brain, was noted and burden assessed by total count. We used the gradient-recalled echo scans and the simplified Brain Observer Microbleeds Scale^22^ to count microbleeds. Cerebral atrophy was defined as enlargement of the ventricles (deep atrophy) and enlargement of the sulci (superficial atrophy) and scored accordingly by classifying each participant on a validated 6-point scale^23^ against a template of normal reference brains. Three analysts did the rating; inter-rater agreement (κ) was 0.66 to 1.0.

### Total SVD Score

A total SVD score (range 0–4)^24,25^ was calculated from individual imaging features by awarding points as follows: 1 for any lacunes, 1 for any microbleeds, 1 for moderate-to-severe PVS in the basal ganglia (grade 2–4), and 1 for WMHs (deep tissue: Fazekas score 2 or 3 and/or periventricular: Fazekas score 3). The total SVD score correlated with both WMH volume (see below) (*r*=0.61; *P*<0.0001) and summed total Fazekas score (*r*=0.65; *P*<0.0001).

### Volumetric Imaging Measures

Intracranial volume (ICV), cerebrospinal fluid, and brain tissue volume (BTV) were measured using Analyze 7.5 (http://analyzedirect.com). The methods have been validated extensively.^21^ The BTV:ICV ratio was also used as a volumetric measure of atrophy; lower values reflecting lower brain tissue volume. The BTV:ICV ratio correlated with the atrophy scores (deep *r*=−0.72; *P*<0.0001 and superficial *r*=−0.74; *P*<0.0001). A volumetric measure of WMH (in mL) was calculated using validated in-house software (MCMXXXVI, available from http://sourceforge.net/projects/bric1936/?source=directory), as described previously.^26^ The effect of head size was corrected for by dividing WMH volume by ICV. The WMH volume correlated with the total Fazekas score (*r*=0.83; *P*<0.0001).

### Plasma Biomarkers

Participants had blood drawn on the day of MRI scanning to assess levels of the cytokine interleukin-6, endothelial dysfunction (von Willebrand Factor antigen and 2 measures of von Willebrand Factor activity: factor VIIIc and ristocetin cofactor), endothelial toxicity (homocysteine), cholesterol (total, high-density lipoprotein [HDL], and low-density lipoprotein), and antiphospholipid antibodies (anticardiolipin IgG and IgM). Blood samples were analyzed in a fully accredited, major NHS laboratory (http://www.edinburghlabmed.co.uk) that handles thousands of samples per day. Patients also had blood tests at recent clinic visits including that for SLE disease activity (C3, C4, and anti–double- stranded DNA) and for routine inflammatory markers (CRP and erythrocyte sedimentation rate [ESR]).

### Statistical Analysis

We tested the association between total SVD score and the Fazekas score and, separately, the WMH volume. We compared age and sex pairwise (SLE to healthy controls; SLE to stroke) by Student *t* test and χ^2^ test, respectively. The individual features of SVD were compared for differences across the 3 subject groups by the Kruskal–Wallis test (the nonparametric equivalent of ANOVA); a post-test multiple comparisons test was used to identify the source of the difference.

We used ordinal logistic regression to test for associations between the total SVD score (range 0–4) and vascular risk factors (age, body mass index, cholesterol, and hypertension but not diabetes mellitus because no SLE patients had diabetes mellitus); plasma biomarkers of inflammation (interleukin-6, ESR, and CRP); endothelial dysfunction (von Willebrand Factor) and toxicity (homocysteine); rheumatology scores (SLEDAI, BILAG, and SLICC); SLE disease duration; plasma markers of SLE activity (C3, C4, and anti–double-stranded DNA); antiphospholipid antibodies; and brain atrophy. Results are presented as odds ratios (ORs) with 95% confidence intervals (95% CIs). For transparency, we report all results regardless of the *P* value because this aids interpretation of the entire study, and we did not adjust the *P* values for multiple comparisons.^27^ A *P* value of <0.05 was considered significant. All analyses were performed in R, version 3.0.1 (http://www.r-project.org/).^28^

## Results

### Subjects

Of 55 consecutive patients with SLE, 51 (mean age: 48.8 years; SD: 14.3 years) agreed to participate, including 47 women (92%), and were compared with 51 healthy controls (39 women [76%; *P*=0.06)) and 51 stroke patients (47 women; *P*=0.99). Of the 4 SLE patients who did not participate, 2 had previous MRI claustrophobia and 2 did not give a reason. Clinical data are given in Table 1 and blood results in Table II in the online-only Data Supplement. Healthy controls were of similar age (mean age: 44.9 years; SD: 11.1 years; *P*=0.12), whereas the stroke patients were on-average 6 years older (mean age: 55.3 years; SD: 8.9 years; *P*=0.008) than the SLE patients. Four SLE patients had NPSLE (monitored by neurology, but none were being treated for active central nervous system disease), 6 were current smokers, 9 had hypertension, none had diabetes mellitus, and 1 had a previous ischemic stroke. Eighteen were prescribed steroids at the time of assessment. There were significantly more smokers and hypertensives in the stroke group. The inflammatory markers ESR and CRP were raised in 22 out of 49 (45%) and 17 out of 45 (38%) of SLE patients versus these tests’ normal reference ranges. Homocysteine was raised in 37 out of 45 (82%) SLE patients.

**Table 1.**
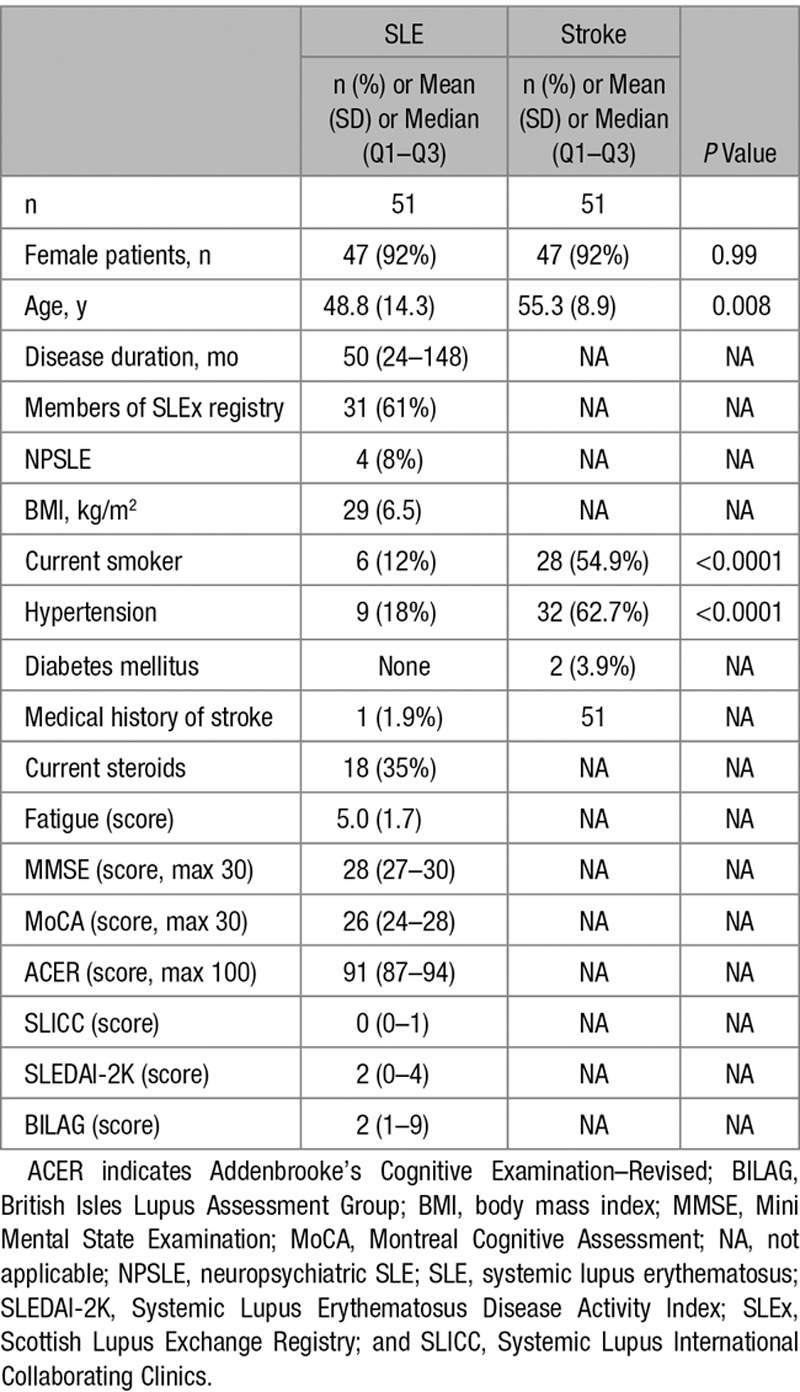
Subject Characteristics

### WMH, PVS, Lacunes, and Microbleeds in SLE

Periventricular and deep WMHs were seen in 49 out of 51 (96%) and 36 out of 51 (70%) SLE patients, respectively. All SLE patients had visible PVS. Lacunes were seen in 5 (10%) and microbleeds in 2 (4%) SLE patients.

### SVD Imaging Biomarkers Versus Healthy Controls and Stroke Patients

Compared with healthy controls, SLE patients had a greater total SVD score (Table 2) sustained across each 10-year age band (Figure), including more deep but not periventricular WMHs. Compared with stroke patients, the SLE patients also had a higher total SVD score, mostly because of having more PVS. SLE patients had more superficial, but not deep, atrophy versus healthy controls. There was no difference in either deep or superficial atrophy score between SLE and stroke patients.

**Table 2.**
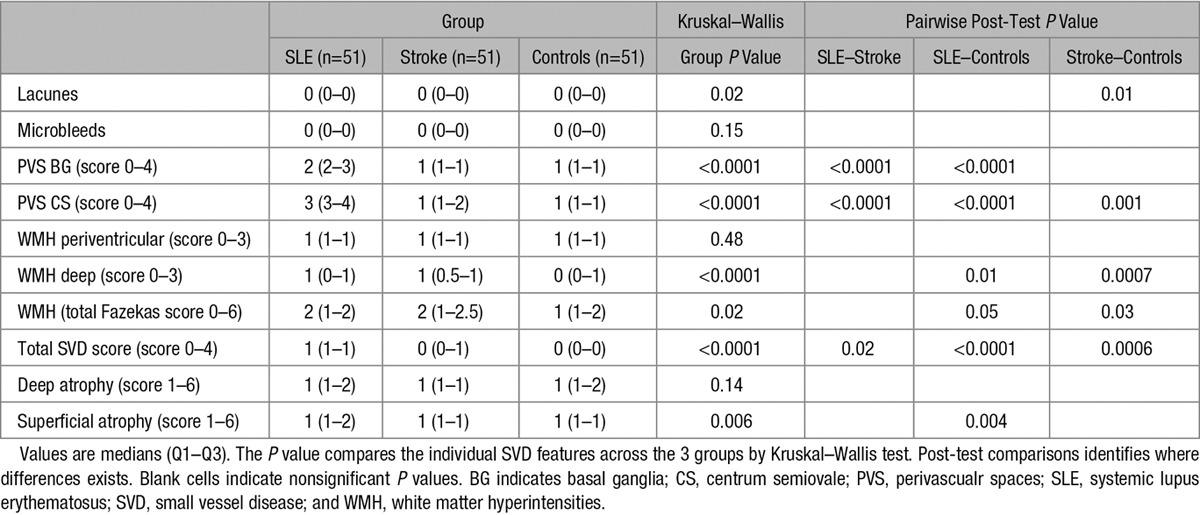
Imaging Biomarkers of SVD in SLE Patients, Healthy Controls, and Stroke Patients

**Figure.**
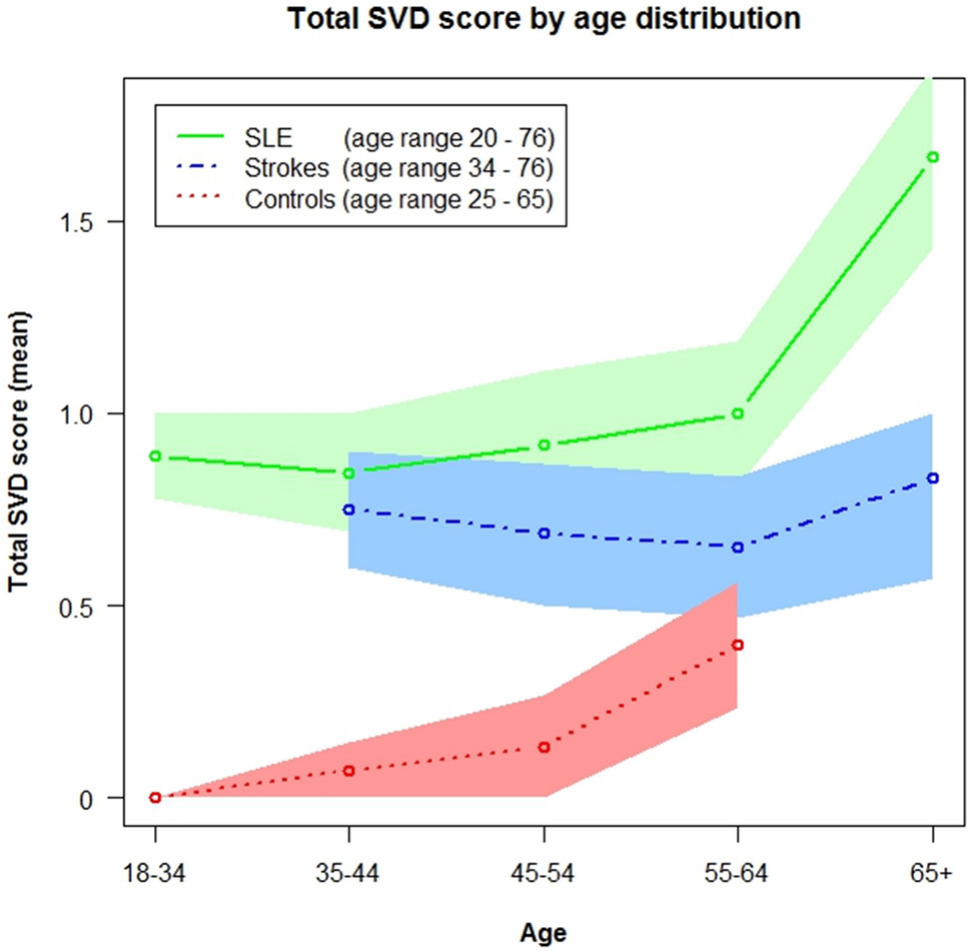
Total small vessel disease (SVD) score by age distribution in systemic lupus erythematosus (SLE), stroke, and healthy controls.

### Association Between Total SVD Score and Other Variables

In SLE, the SVD score was associated in univariate analyses with age (OR, 1.05; 95% CI, 1.01–1.09), hypertension (OR, 1.82; 95% CI, 1.13–2.93), higher levels of mean diffusivity (OR, 2.58; 95% CI, 1.32–5.06), and lower levels of fractional anisotropy (OR, 0.42; 95% CI, 0.22–0.80). The association with hypertension did not remain after adjusting for age (Table III in the online-only Data Supplement). Higher SVD burden was inversely associated with fatigue but not when adjusted for age. The total SVD score was not associated with SLE activity (by SLE activity scoring tools or blood markers of activity), accumulated damage (SLICC), SLE disease duration, inflammatory markers, or cognitive variables. More PVS in the centrum semiovale were associated with higher levels of HDL cholesterol (OR, 14.88; CI, 2.76–80.09; Table IV in the online-only Data Supplement), which remained significant after adjusting for age and body mass index (OR, 16.99; CI, 2.98–96.66). No other individual SVD feature showed significant associations with other variables (vascular risk factors, SLE activity, or blood markers).

### Neuropsychiatric Systemic Lupus Erythematosus

Patients diagnosed with NPSLE (n=4) had more deep WMH compared with SLE (*P*=0.04; Table V in the online-only Data Supplement), but data were limited.

## Discussion

We show that patients with SLE have more SVD neuroimaging markers, notably PVS and deep WMH, than sex- and age-matched healthy controls and more PVS than patients with minor stroke patients from the same health region. Deep WMH were worse in 4 patients with NPSLE, but the number of patients with NPSLE in our study was small that limits generalizability. Our patients were not selected on the basis of neurological involvement, and only 4 out of 51 were diagnosed with NPSLE. Despite the stroke patients being slightly older, with far more smokers and hypertensives, the SLE patients had more PVS and an equal burden of WMH. The higher burden of PVS in SLE patients, in the absence of vascular risk factors, is consistent with the known association between PVS and plasma markers of inflammation in patients with SVD and suggests that inflammation may be associated with subclinical microvascular brain damage in SLE patients. However, although the present study design demonstrates an association between SVD imaging features and a systemic inflammatory disease, it cannot identify the nature of the inflammation, whether local or vascular/systemic. The study was exploratory, and a larger study, with more power to detect differences between groups while also accounting for traditional risk factors, is warranted. The higher burden of SVD also provides a possible explanation for increased stroke risk; a hypothesis that should be investigated.

A limitation of the current cross-sectional study design is that it cannot ascertain a temporal relationship for the SVD markers within the SLE group. A longitudinal study, ideally from initial presentation with SLE, would be required to determine the pattern of SVD development in relation to SLE activity and duration.

Several cross-sectional studies (reviewed in Wiseman et al^1^), including ≈1200 SLE patients, reported features of SVD, such as WMHs and atrophy, but many of these studies focused on NPSLE patients, few compared SLE to healthy controls, none compared SLE to minor stroke patients, which allows for comparison with clinically overt SVD, and none included the range of SVD features assessed here with validated scoring tools. Longitudinally, a 20-year MRI follow-up study showed increased number and volume of WMHs and brain volume loss in most of 30 SLE patients studied, but may have reflected mainly ageing effects.^29^ In a shorter follow-up study of 75 SLE patients, predictors of new or increased WMH included antiphospholipid antibodies, SLE damage scores, and higher dose of corticosteroids (possibly a marker of higher disease activity), and there was more gray and white matter volume loss versus controls.^30,31^

PVS on neuroimaging are associated with inflammatory activity.^32^ PVS were associated with the inflammatory marker CRP in a large cohort (n=634) of community-dwelling older people (ß=0.12; *P*=0.048).^5^ In our study, the total SVD score and PVS were not associated with any blood measure of inflammation (ESR, CRP, interleukin-6) or with clinical SLE disease activity or disease burden score, but this may reflect the small sample. However, we note that ≈40% of patients had raised ESR and CRP. Moreover, our cross-sectional study design did not permit us to associate inflammatory flares over time with the evolution, or not, of PVS or WMH. We did not select patients on the basis of SLE activity and cannot exclude the possibility that blood markers of inflammation (eg, captured during a flare-up) will associate with PVS in a larger or longitudinal study. Chung et al^33^ also found no association between systemic inflammation (measured using a novel marker, GlycA) and SLE activity, despite the presence of systemic inflammation.

Perivascular inflammation of the small cerebral vessels is a prominent finding in SLE^34^ at autopsy and in sporadic SVD.^4^ Some studies have noted PVS^35,36^ on brain imaging in SLE patients (n=122), but data are limited, and none compared the total load with a non-SLE comparator group. In a recent, but smaller (n=11), postmortem study of vascular changes in SLE, one third of subjects had microthromboemboli, glial hyperplasia, neuronal loss, microaneurysms, lacunar infarcts and microbleeds, which correlated with neuroimaging, including recent subcortical infarcts, lacunes, WMHs, and atrophy; stroke and cognitive impairment were more frequent findings among these patients compared with the SLE patients who did not have histological evidence of SVD.^37^

We note an association between higher levels of HDL cholesterol and more PVS in the centrum semiovale, which remained significant after adjusting for age and body mass index. High HDL cholesterol is traditionally considered protective against cardiovascular diseases including ischemic stroke, but a recent meta-analysis showed that drugs designed to boost HDL did not improve cardiovascular outcomes.^38^ Additionally, a gene variation in some people impairs HDL uptake, making them susceptible to cardiovascular disease despite high HDL.^39^ In a cohort study^40^ of 210 SLE patients followed for 29 months, functional HDL (a novel marker of inflammation) was associated with carotid plaques (OR, 9.1; 95% CI, 3.3–24.6). Meanwhile, higher HDL is associated with increased risk of hemorrhagic stroke in the general population (relative risk, 1.17; 95% CI, 1.02–1.35; 7960 strokes, 1.4 million participants).^41^ The reason why cholesterol might relate to PVS in SLE is unknown and could be spurious given our study’s lack of power, but HDL can become dysfunctional resulting in inflammation^42,43^ and endothelial dysfunction.^44^

Our analysis provides support for the concept of a total SVD score^24,25^ as a simple surrogate marker for total brain damage because of SVD. The association of deep and superficial atrophy on univariate analysis, but not in adjusted analyses, is in agreement with Staals et al^24^ and suggests that atrophy should remain complementary, but not core, in the SVD score as atrophy coassociates with age. Our volume measure of atrophy (the BTV:ICV ratio) was also associated with total SVD burden. Hypertension (dichotomized [data not shown] and on a 6-point scale) was also associated with the total SVD score, but unlike Staals et al,^24^ smoking (current or ever) was not, although our study was underpowered and only 12% of our patients smoked compared with one third of theirs.

We did not collect concomitant renal pathology data. Other limitations include a potential source of bias as the healthy controls were recruited from the community by advertising and required to be without known vascular risk factors such as hypertension or diabetes mellitus. We may, thus, have influenced the associations of SVD burden in the stroke and SLE patients, although few of the SLE patients had hypertension or diabetes mellitus. We tried to avoid selection bias in the SLE group by recruiting consecutively and hence included SLE patients with a range of disease durations and severities. A much larger study, sufficiently powered, is now justified to assess the influence of traditional vascular risk factors and the effects of treatments on SVD burden in SLE. Additionally, larger longitudinal studies are needed to fully appreciate the significance of SVD in SLE, for example, to elucidate the contribution of SLE activity, diet and lifestyle, SLE treatments, or some other variable in causing accelerated brain damage in these patients.

## Sources of Funding

This study was funded by Lupus UK. Data relating to the stroke patients was funded by the Wellcome Trust (WT088134/Z/09/A). Data relating to the controls was funded by the NIH (R01 EB004155-03).

## Disclosures

None.

## Supplementary Material
