# Supplementary material for: Cerebral Small Vessel Disease Burden Is Increased in Systemic Lupus Erythematosus
Source: Stroke. 2016 Oct 25;47(11):2722–8. doi: 10.1161/STROKEAHA.116.014330 (PMC5079231; doi:10.1161/STROKEAHA.116.014330)
Supplement: Supplementary file 1 [file str-47-2722-s001.pdf]

# ONLINE SUPPLEMENT

**Title:**

Cerebral small vessel disease burden is increased in systemic lupus erythematosus.


**Authors:**

Stewart J. Wiseman[1], Mark E. Bastin[1], Charlotte L. Jardine[1], Gayle Barclay[1], Iona F. Hamilton[1], Elaine Sandeman[1], David Hunt[1], E. Nicole Amft[2], Susan Thomson[3], Jill F.F. Belch[3], Stuart H. Ralston[4], Joanna M. Wardlaw[1].

**Affiliation:**

[1] Centre for Clinical Brain Sciences, University of Edinburgh, UK
[2] Department of Rheumatology, Western General Hospital, Edinburgh, UK
[3] Division of Cardiovascular and Diabetes Medicine, University of Dundee, UK
[4] Centre for Genomic and Experimental Medicine, University of Edinburgh, UK

**Correspondence:**

Joanna Wardlaw, CCBS, Chancellor's Building, Royal Infirmary of Edinburgh, 49 Little France Crescent, Edinburgh, EH16 4SB. E-mail: Joanna.Wardlaw@ed.ac.uk Tel: 0131 242 6200. Fax: 0131 242 6210.

**Table I** Scanning parameters

56 slices, 24 FOV, Top to bottom (start 1 slice above brain tissue), Straight axials (not AC-PC),

DTI = set fft_xsize + fft_ysize =128 and note locations.

| SEQ. NUMBER | 1 | 2 | 3 | 4 | 5 | 6 | 7 | 8 | 9 |
|---|---|---|---|---|---|---|---|---|---|
| **SEQUENCE** | Loc | FLAIR | T2 | GRE | Sag T2 Cube | 3D IR PREP | DTI (32 directions) | FSPGR 2 | FSPGR 12 |
| **ORIENTATION** | | AX | AX | AX | SAG | COR | AX | AX | AX |
| **TE** | | 140 | 102 | 14 | | MIN/FUL | MIN | MIN FULL | MIN FULL |
| **TR** | | 9400 | 8750 | 1420 | 3000 | | 13750 | | |
| **TI/prep time** | | 2350 | | | | 500 | | | |
| **FOV** | | 24 | 24 | 24 | 24 | 24 | 24 | 24 | 24 |
| **SLICE THICK.** | | 5 | 2.5 | 2.5 | 1 | 1.3 | 2.5 | 2.5 | 2.5 |
| **SLICE GAP** | | 0 | 0 | 0 | 0 | 0 | 0 | 0 | 0 |
| **Acq. MATRIX** | | 384 x 256 | 384 x 384 | 384 x 256 | 320 x 320 | 192 x 192 | 96 x 96 | 128 x 128 | 128 x 128 |
| **Padded (R x C)** | | 512 x 512 | 512 x 512 | 512 x 512 | 512 x 512 | 256 x 256 | 128 x 128 | 256 x 256 | 256 x 256 |
| **Pixel width** | | 0.47 | 0.47 | 0.47 | 0.47 | 0.94 | 1.875 | 0.94 | 0.94 |
| **Pixel height** | | 0.47 | 0.47 | 0.47 | 0.47 | 0.94 | 1.875 | 0.94 | 0.94 |
| **Voxel depth** | | 5 | 2.5 | 2.5 | 1 | 1.3 | 2.5 | 2.5 | 2.5 |
| **Resolution** | | 2.133 pix/mm | 2.133 pix/mm | 2.133 pix/mm | 2.133 pix/mm | 1.067 pix/mm | 0.533 pix/mm | 1.067 pix/mm | 1.067 pix/mm |
| **PHASE FOV** | | | 1 | 1 | | 1 | 1 | 1 | 1 |
| **FREQ. DIR** | | AP | AP | AP | | SI | RL | AP | AP |
| **BAND WIDTH** | | 15.63 | 20.83 | 12.50 | | 15.63 | | 27.78 | 27.78 |
| **NEX** | | 1 | 1 | 1 | 1 | 1 | 1 | 1 | 1 |
| **FLIP ANGLE** | | | | 20 | | 8 | | 2 | 12 |
| **NO. SLICES** | | 28 | 56 | 56 | 1 SLAB (180 locs) | 1 SLAB (160 locs) | 56 | 1 SLAB (62 locs) | 1 SLAB (62 locs) |
| **TIME of ACQ.** | 0:10 | 6:16 | 5:59 | 6:09 | 5:13 | 8:12 | 8:15 | 00:49 | 00:49 |

Brain Research Imaging Centre, University of Edinburgh. 2104. www.bric.ed.ac.uk

**Table II** Blood results for 51 SLE patients

| | **N (%) or mean ± SD or median (Q1–Q3)** | **Reference range** |
|---|---|---|
| **Rheumatological** | | |
| C3 (mg / dL) (n=47) | 1.2 ± 0.32 | 0.81–1.57 |
| C4 (mg / dL) (n=47) | 0.19 ± 0.09 | 0.13–0.39 |
| Anti-ds-DNA (IU / mL) (n=47) | 15 (8.5–33) | 0–20 |
| **Inflammatory** | | |
| ESR (mm / hr) (n=49) | 13 (6–21) | 3–15 |
| CRP (mg / L) (n=46) | 2 (1–8) | 0–5 |
| IL-6 (pg / mL) (n=40) | 1.72 (1.12–2.37) | |
| IFN (RQ value) (n=25) | 6.7 (0.60–18.5) | |
| **Endothelial dysfunction** | | |
| vWF Ag (IU / mL) (n=46) | 1.71 ± 0.66 | 0.42–1.22 |
| vWF fVIIIc (IU / mL) (n=46) | 1.38 ± 0.45 | 0.5–1.5 |
| vWF RCOF (IU / mL) (n=46) | 1.37 ± 0.41 | 0.42–1.22 |
| Homocysteine (umol / L) (n=45) | 17 (15–21) | 0–20 |
| **Lipids** | | |
| Total cholesterol (mmol / L) (n=49) | 5.02 ± 0.95 | <5.2 |
| HDL cholesterol (mmol / L) (n=47) | 1.42 ± 0.41 | >1 |
| LDL cholesterol (mmol / L) (n=46) | 2.96 ± 0.84 | <3.3 |
| **Antibodies** | | |
| Anti-cardiolipin IgG (GPL) (n=50) | 3.35 (2.20–5.60) | 0–13.3 |
| Anti-cardiolipin IgM (MPL) (n=50) | 1.65 (1.10–3.30) | 0–9.8 |
| Lupus anticoagulant (n=47) | 5 positive | Positive/Negative |

Anti-ds-DNA = anti-double stranded DNA, CRP = C-reactive protein, ESR = erythrocyte sedimentation rate, HDL = high density lipoprtein, IFN = interferon, IgG = immunoglobulin isotype G, IgM = immunoglobulin isotype M, IL-6 = interleukin-6, LDL = low density lipoprotein, vWF Ag = von Willebrand Factor antigen, vWF fVIIIc = von Willebrand Factor factor VIIIc, vWF RCOF = von Willebrand Factor ristocetin co-factor

**Table III** Univariate and multivariable associations between total SVD score and other variables in SLE patients

| | Unadjusted | | Age adjusted | | Adjusted for age and SLE disease duration | |
|---|---|---|---|---|---|---|
| | **OR** | **95%CI** | **OR** | **95%CI** | **OR** | **95%CI** |
| **Vascular risk factors** | | | | | | |
| Age (years) | 1.05 | 1.01 – 1.09 * | -- | -- | 1.05 | 1.00 – 1.10 * |
| Hypertension (classified) | 1.82 | 1.13 – 2.93 * | 1.58 | 0.95 – 2.63 | 1.58 | 0.95 – 2.63 |
| BMI (kg / $m^2$) | 0.97 | 0.89 – 1.06 | 0.95 | 0.87 – 1.04 | 0.95 | 0.87 – 1.04 |
| Disease duration (months) | 1.00 | 0.99 – 1.01 | 1.00 | 0.99 – 1.00 | -- | -- |
| Current smoker (Yes/No) | 0.91 | 0.15 – 5.29 | 1.19 | 0.19 – 7.32 | 1.22 | 0.19 – 7.55 |
| Ever smoked (Yes/No) | 2.06 | 0.63 – 6.80 | 2.00 | 0.67 – 6.62 | 2.01 | 0.60 – 6.76 |
| Steroids (Yes/No) | 0.63 | 0.18 – 2.16 | 0.62 | 0.18 – 2.14 | 0.62 | 0.18 – 2.13 |
| **Neurological** | | | | | | |
| Fatigue | 0.68 | 0.47 – 0.98 * | 1.04 | 0.99 – 1.08 | 1.00 | 0.99 – 1.00 |
| Anxiety | 0.97 | 0.87 – 1.08 | 1.02 | 0.90 – 1.14 | 1.02 | 0.90 – 1.14 |
| Depression | 0.97 | 0.85 – 1.09 | 1.01 | 0.88 – 1.15 | 1.01 | 0.88 – 1.15 |
| MMSE | 0.94 | 0.63 – 1.38 | 1.05 | 0.69 – 1.58 | 1.07 | 0.69 – 1.67 |
| MoCA | 1.06 | 0.89 – 1.26 | 1.11 | 0.92 – 1.33 | 1.12 | 0.93 – 1.36 |
| ACER | 1.01 | 0.94 – 1.09 | 1.02 | 0.95 – 1.11 | 1.03 | 0.95 – 1.12 |
| **Rheumatology scores** | | | | | | |
| SLEDAI-2K | 1.01 | 0.78 – 1.30 | 1.01 | 0.78 – 1.31 | 1.01 | 0.78 – 1.31 |
| BILAG | 0.95 | 0.85 – 1.07 | 0.98 | 0.87 – 1.11 | 0.99 | 0.87 – 1.11 |
| SLICC | 1.14 | 0.68 – 1.91 | 0.93 | 0.53 – 1.62 | 0.88 | 0.48 – 1.65 |
| **DTI biomarkers** | | | | | | |
| MD across 12 tracts (n=47) | 2.58 | 1.32 – 5.06 † | 2.32 | 1.16 – 4.64 * | 2.53 | 1.22 – 5.22 * |
| FA across 12 tracts (n=47) | 0.42 | 0.22 – 0.80 † | 0.47 | 0.24 – 0.93 * | 0.43 | 0.21 – 0.88 * |
| **Bloods** | | | | | | |
| C3 (mg / dL) (n=47) | 1.06 | 0.15 – 7.56 | 1.06 | 0.14 – 8.11 | 1.14 | 0.14 – 8.93 |
| C4 (mg / dL) (n=47) | 1.07 | 0.52 – 2.20 | 1.14 | 0.55 – 2.35 | 1.17 | 0.56 – 2.47 |
| Anti-ds-DNA (IU / mL) (n=47) | 0.99 | 0.98 – 1.01 | 0.99 | 0.98 – 1.00 | 0.99 | 0.98 – 1.01 |
| ESR (mm / hr) (n=49) | 1.03 | 0.99 – 1.06 | 1.02 | 0.98 – 1.06 | 1.02 | 0.98 – 1.06 |
| CRP (mg / L) (n=45) | 0.99 | 0.92 – 1.08 | 0.99 | 0.92 – 1.08 | 1.00 | 0.92 – 1.08 |
| IL-6 (pg / mL) (n=40) | 0.99 | 0.71 – 1.40 | 1.02 | 0.72 – 1.43 | 1.02 | 0.72 – 1.43 |
| IFN (RQ value) (n=24) | 1.04 | 0.94 – 1.14 | 1.04 | 0.95 – 1.14 | 1.04 | 0.93 – 1.16 |
| vWF Ag (IU / mL) (n=46) | 1.21 | 0.48 – 3.00 | 0.88 | 0.33 – 2.30 | 0.87 | 0.33 – 2.31 |
| vWF F8c (IU / mL) (n=46) | 2.50 | 0.66 – 9.45 | 2.30 | 0.62 – 8.57 | 2.31 | 0.62 – 8.61 |
| vWF RCOF (IU / mL) (n=46) | 2.00 | 0.46 – 8.56 | 1.24 | 0.26 – 5.83 | 1.25 | 0.26 – 5.91 |
| Homocysteine (umol / L) (n=45) | 1.03 | 0.94 – 1.12 | 1.01 | 0.92 – 1.11 | 1.01 | 0.92 – 1.11 |
| Tot cholesterol (mmol / L) (n=49) | 1.55 | 0.84 – 2.87 | 1.37 | 0.72 – 2.59 | 1.38 | 0.73 – 2.62 |
| HDL cholesterol (mmol / L) (n=47) | 1.35 | 0.32 – 5.74 | 1.41 | 0.33 – 6.04 | 1.39 | 0.32 – 6.02 |
| LDL cholesterol (mmol / L) (n=46) | 1.72 | 0.85 – 3.49 | 1.47 | 0.71 – 3.01 | 1.50 | 0.71 – 3.14 |
| Anti-cardiolipin IgG (GPL) (n=50) | 0.92 | 0.77 – 1.09 | 0.93 | 0.78 – 1.11 | 0.93 | 0.78 – 1.10 |
| Anti-cardiolipin IgM (MPL) (n=50) | 1.00 | 0.95 – 1.06 | 0.98 | 0.93 – 1.04 | 0.98 | 0.93 – 1.04 |
| Lupus anticoagulant (n=47) | 0.42 | 0.06 – 2.73 | 0.30 | 0.04 – 2.18 | 0.30 | 0.04 – 2.15 |

* $p<0.05$, † $p<0.01$

ACER = Addenbrooke's Cognitive Examination – Revised, BMI = body mass index, BILAG = British Isles Lupus Assessment Group, BTV = brain tissue volume, CRP = C-reactive protein, DTI = diffusion tensor imaging, ESR = erythrocyte sedimentation rate, FA = fractional anisotropy, HDL = high density lipoprotein, LDL = low density lipoprotein, MD = mean difussivity, MMSE = Mini Mental State Examination, MoCA = Montreal Cognitive Assessment, IFN = interferon beta, IL-6 = interleukin-6, ICV = intracranial volume, SLEDAI-2K = systemic lupus erythematosus Disease Activity Index, SLICC = Systemic Lupus International Collaborating Clinics, vWF Ag = von Willebrand Factor antigen, vWF F8c = von Willebrand Factor VIII, vWF RCOF = von Willebrand Factor ristocen co-factor

**Table IV** Unadjusted univariable associations between individual SVD features and other variables in SLE patients

| | PVS BG | | PVS CS | | WMH (Total Fazekas) | |
|---|---|---|---|---|---|---|
| | **OR** | **95%CI** | **OR** | **95%CI** | **OR** | **95%CI** |
| **Vascular risk factors** | | | | | | |
| Age (years) | 1.02 | 0.98 – 1.06 | 1.03 | 0.99 – 1.07 | 1.13 | 1.07 – 1.20 ‡ |
| Hypertension (classified) | 1.36 | 0.90 – 2.05 | 1.25 | 0.84 – 1.86 | 1.88 | 1.20 – 2.94 † |
| BMI (kg / m$^2$) | 0.97 | 0.89 – 1.05 | 0.98 | 0.91 – 1.07 | 1.03 | 0.95 – 1.25 |
| Disease duration (months) | 1.00 | 0.99 – 1.00 | 1.00 | 0.99 – 1.00 | 1.00 | 0.99 – 1.00 |
| Current smoker (Yes/No) | 0.87 | 0.19 – 3.92 | 0.50 | 0.11 – 2.34 | 1.87 | 0.37 – 9.40 |
| Ever smoked (Yes/No) | 0.82 | 0.30 – 2.23 | 0.77 | 0.27 – 2.18 | 2.63 | 0.87 – 7.89 |
| Steroids (Yes/No) | 0.48 | 0.17 – 1.41 | 0.74 | 0.25 – 2.24 | 1.44 | 0.47 – 4.36 |
| **Rheumatology scores** | | | | | | |
| SLEDAI | 1.10 | 0.88 – 1.37 | 1.04 | 0.80 – 1.35 | 1.00 | 0.78 – 1.29 |
| BILAG | 0.96 | 0.86 – 1.07 | 0.93 | 0.84 – 1.04 | 0.96 | 0.86 – 1.07 |
| SLICC | 0.86 | 0.55 – 1.35 | 0.92 | 0.57 – 1.49 | 1.42 | 0.88 – 2.28 |
| **Bloods** | | | | | | |
| C3 (mg / dL) (n=47) | 0.35 | 0.06 – 1.98 | 1.08 | 0.22 – 5.29 | 0.70 | 0.13 – 3.71 |
| C4 (mg / dL) (n=47) | 0.85 | 0.45 – 1.62 | 1.16 | 0.64 – 2.09 | 0.79 | 0.42 – 1.49 |
| Anti-ds-DNA (IU / mL) (n=47) | 1.00 | 0.99 – 1.02 | 1.00 | 0.99 – 1.01 | 1.00 | 0.99 – 1.01 |
| ESR (mm / hr) (n=49) | 1.02 | 0.98 – 1.05 | 0.99 | 0.96 – 1.02 | 1.03 | 0.99 – 1.06 |
| CRP (mg / L) (n=45) | 1.00 | 0.99 – 1.03 | 1.02 | 0.98 – 1.06 | 1.02 | 0.99 – 1.04 |
| IL-6 (pg / mL) (n=40) | 1.04 | 0.79 – 1.37 | 0.94 | 0.71 – 1.24 | 0.98 | 0.74 – 1.29 |
| IFN (RQ value) (n=24) | 0.93 | 0.86 – 1.00 | 0.97 | 0.91 – 1.05 | 0.98 | 0.90 – 1.06 |
| vWF Ag (IU / mL) (n=46) | 0.96 | 0.43 – 2.17 | 1.56 | 0.67 – 3.60 | 1.30 | 0.56 – 2.99 |
| vWF F8c (IU / mL) (n=46) | 1.39 | 0.40 – 4.84 | 2.58 | 0.69 – 9.64 | 1.89 | 0.54 – 6.63 |
| vWF RCOF (IU / mL) (n=46) | 1.07 | 0.29 – 3.89 | 1.62 | 0.42 – 6.21 | 1.34 | 0.31 – 5.76 |
| Homocysteine (umol / L) (n=45) | 0.97 | 0.89 – 1.04 | 1.02 | 0.94 – 1.11 | 1.07 | 0.98 – 1.16 |
| Tot cholesterol (mmol / L) (n=49) | 1.75 | 0.98 – 3.12 | 1.40 | 0.80 – 2.46 | 1.31 | 0.73 – 2.35 |
| HDL cholesterol (mmol / L) (n=47) | 3.30 | 0.86 – 12.6 | 14.8 | 2.76 – 80.0 ‡ | 0.95 | 0.23 – 3.99 |
| LDL cholesterol (mmol / L) (n=46) | 1.89 | 0.96 – 3.71 | 1.11 | 0.58 – 2.12 | 1.38 | 0.70 – 2.73 |
| Anti-cardiolipin IgG (GPL) (n=50) | 1.00 | 0.86 – 1.16 | 0.94 | 0.80 – 1.09 | 0.88 | 0.75 – 1.04 |
| Anti-cardiolipin IgM (MPL) (n=50) | 1.02 | 0.97 – 1.08 | 1.02 | 0.97 – 1.07 | 0.99 | 0.94 – 1.04 |
| Lupus anticoagulant (n=47) | 0.79 | 0.14 – 4.67 | 1.23 | 0.22 – 6.84 | 0.60 | 0.11 – 3.22 |

* p<0.05, † p<0.01 ‡ p<0.0001

BMI = body mass index, BILAG = British Isles Lupus Assessment Group, BTV = brain tissue volume, CI = confidence interval, CRP = C-reactive protein, DTI = diffusion tensor imaging, ESR = erythrocyte sedimentation rate, HDL = high density lipoprotein, LDL = low density lipoprotein, IFN = interferon beta, IL-6 = interleukin-6, ICV = intracranial volume, OR = odds ratio, PVS BG = perivascular spaces in basal ganglia, PVS BG = perivascular spaces in centrum semiovale, SLEDAI = systemic lupus erythematosus Disease Activity Index, SLICC = Systemic Lupus International Collaborating Clinics, vWF Ag = von Willebrand Factor antigen, vWF F8c = von Willebrand Factor VIII, vWF RCOF = von Willebrand Factor ristocen co-factor, WMH = white matter hyperintensties

**Table V** Imaging biomarkers of SVD in SLE and NPSLE patients

| | SLE | NPSLE | |
|---|---|---|---|
| | **Median (Q1–Q3)** | **Median (Q1–Q3)** | **p value** |
| N | 47 | 4 | |
| Lacunes | 0 (0–0) | 0 (0–0) | 0.52 |
| Microbleeds | 0 (0–0) | 0 (0–0) | 0.71 |
| PVS BG (score 0–4) | 2 (2–3) | 2.5 (1.5–3.0) | 0.76 |
| PVS CS (score 0–4) | 3 (3–4) | 3.0 (1.5–4.0) | 0.72 |
| WMH periventricular (score 0–3) | 1 (1–1) | 1.5 (0.5–2.5) | 0.43 |
| WMH deep (score 0–3) | 1 (0–1) | 1.5 (1.0–2.5) | 0.04 * |
| WMH (total Fazekas score 0–6) | 2 (1–2) | 3.0 (1.5–5.0) | 0.22 |
| **Total SVD score (score 0–4)** | **1 (1–2)** | **1.5 (1.0–3.5)** | **0.43** |
| Deep atrophy (score 1–6) | 1 (1–2) | 1.5 (1.0–2.5) | 0.49 |
| Superficial atrophy (score 1–6) | 1 (1–1) | 1.5 (0.5–2.0) | 0.59 |

* p<0.05
BG = basal ganglia, CS = centrum semiovale, NPSLE = neurpsychiatric SLE, PVS = perivascualr spaces, SVD = small vessel disease, SLE = systemic lupus erythematosus, WMH = white matter hyperintensities.

## Medications

Fifty of 51 SLE patients were taking one or more regular medications for SLE at time of MR scanning, including non-steroidal anti-inflammatory drugs (n=2 SLE patients); anti-malarials (n=40); immunosupressants (n=30); corticosteroids (n=18); and biological therapy (n=1). Additioanlly, one patient was on warfarin, several were on alendronic acid for osteoperosis and many were taking pain killers as required. We cross-checked these self-reported medications with records from rheumatology / the NHS.
